# Entwicklung konkreter Handlungsoptionen für die sichere Öffnung und den Betrieb von Freizeitdestinationen unter Pandemiebedingungen

**DOI:** 10.1007/s40664-022-00480-y

**Published:** 2022-10-07

**Authors:** Urs A. Fichtner, Manuela Piotrowsky-Fichtner, Myriel Fichtner, Ann-Kathrin Goßmann, Frederik Weis, Maximilian Weiß, Daniel Steinmann

**Affiliations:** 1 Fichtner*Piotrowsky Projektconsulting, Sparneckerweg 4, 95445 Bayreuth, Deutschland; 2 Institut für Medizinische Biometrie und Statistik – Sektion Versorgungsforschung und Rehabilitationsforschung, Universitätsklinikum Freiburg, Albert-Ludwigs-Universität Freiburg, Hugstetter Straße 49, 79106 Freiburg, Deutschland; 3 Palas GmbH, Greschbachstr. 3b, 76229 Karlsruhe, Deutschland; 4 Betriebsärztlicher Dienst – Universitätsklinikum Freiburg, Breisacher Str. 86b, 79110 Freiburg im Breisgau, Deutschland

**Keywords:** COVID-19, Richtlinie, Hygiene- und Sicherheitskonzept, Freizeiteinrichtungen, Aerosolmessungen, COVID-19, Guideline, Hygiene and safety measures, Recreational facilities, Aerosol measurement

## Abstract

**Hintergrund:**

Die SARS-CoV-2-Pandemie führte weltweit zur Schließung von Freizeit- und Erholungseinrichtungen. Im Rahmen einer vom baden-württembergischen Ministerium für Soziales, Gesundheit und Integration geförderten Modellstudie konnte am Beispiel der Öffnung und des Betriebs eines Freizeitparks unter wissenschaftlicher Begleitung gezeigt werden, wie ein Hygiene- und Sicherheitskonzept in die Praxis erfolgreich umsetzbar ist.

**Ziel:**

Neben der Überprüfung eines möglichen Infektionsgeschehens durch den Besuch des Freizeitparks, zielte das Projekt darauf ab, Handlungsoptionen für die sichere Öffnung und den Betrieb von Freizeiteinrichtungen unter Pandemiebedingungen, die auf andere Destinationen übertragbar sind, zu erarbeiten.

**Material und Methoden:**

Für dieses Projekt wurden verschiedene Datenquellen genutzt: Expertenrunden multidisziplinärer Teams (Betriebswirtschaft, Versorgungsforschung, Soziologie und Medizin), Aerosolmessdaten, Beobachtungsprotokolle, amtliche Infektionszahlen und Daten aus Besucherbefragungen.

**Ergebnisse:**

Die in diesem Projekt entstandenen Handlungsoptionen liefern eine Orientierungshilfe für Betreiber von Freizeiteinrichtungen zur Umsetzung von Maßnahmen, durch welche die Sicherheit des Personals und der Gäste erhöht werden und so den Betrieb der Anlagen unter Pandemiebedingungen ermöglichen.

**Diskussion:**

Diese Studie stellt einen Präzedenzfall am Beispiel eines Freizeitparks in Baden-Württemberg dar, der anderen Einrichtungen als Wegweiser dient. Freizeit- und Tourismusbetriebe sind allerdings einzigartig und Maßnahmen entsprechend schwer direkt übertragbar. Die Handlungsoptionen sollen weiterhin politische Entscheidungsträger in zukünftigen Pandemiesituationen hinsichtlich Maßnahmen zur Schließung, Öffnung und Betrieb solcher Anlagen unterstützen.

**Zusatzmaterial online:**

Zusätzliche Informationen sind in der Online-Version dieses Artikels (10.1007/s40664-022-00480-y) enthalten.

## Abstract (en)

**Background:**

The SARS-CoV‑2 pandemic led to the closure of leisure and recreation facilities worldwide. As part of a model study funded by the Baden-Wuerttemberg Ministry of Social Affairs, Health and Integration, it was possible to demonstrate how a hygiene and safety concept can be successfully implemented in practice using the example of the opening and operation of an amusement park in Baden-Wuerttemberg (Germany) under scientific supervision.

**Objective:**

The aim of the model project was, besides the verification of a possible infection event through a visit to the amusement park, to develop and review a recommended course of action for the safe opening and operation of leisure facilities under pandemic conditions, which can be transferred to other destinations.

**Methods:**

A variety of data sources were used for this project: Recurrent expert rounds of multidisciplinary teams (business administration, healthcare research, sociology and medicine), aerosol measurement data, observation protocols, official infection statistics and interview data from visitor surveys.

**Results:**

The action plan developed in this project provides guidance and recommendations for operators of recreation and leisure facilities to implement measures that enhance staff and guest safety, allowing facilities to operate under pandemic conditions.

**Conclusion:**

This study sets a precedent using the example of a recreational park in Baden-Wuerttemberg (Germany) to serve as a guide for other facilities; however, operations in the leisure and tourism sector are unique and measures are accordingly difficult to transfer directly. The recommended action plan is further intended to support policy makers in future pandemic situations regarding measures to close, open and operate such facilities.

Zur Einschränkung der Ausbreitung von SARS-CoV‑2 erfolgte in Deutschland ab Ende März 2020 eine Erlasskette an Verordnungen und Gesetzen auf Bundes- und Landesebene, die eine Schließung von Gastgewerbe und Freizeiteinrichtungen zur Folge hatte. Zwar wurden angesichts fallender Inzidenzzahlen schrittweise Öffnungen ab Mai 2020 ermöglicht, dies galt allerdings nur unter strengen Hygieneauflagen und Kontaktreduzierung, z. B. durch Einführungen von Besucherobergrenzen. Im Sommer 2020 kündigte sich die zweite Welle der SARS-CoV-2-Pandemie an und eine erneute Schließung der Freizeitindustrie in Deutschland wurde ab 2. November 2020 angeordnet [32]. Der Betriebsstillstand hatte enorme wirtschaftliche Konsequenzen für Betreiber von Freizeitdestinationen und die mittelbar verbundenen Unternehmen, so dass schnell die Entwicklung von Öffnungskonzepten vorangetrieben werden musste [21]. Modellprojekte, wie die kontrollierte Öffnung von Innenstädten (Tübinger Modell) wurden angestoßen, um Lösungsstrategien für den Ausweg aus den behördlich angeordneten Schließungen zu finden [10]. Im ersten Quartal 2021 beschloss das Ministerium für Soziales, Gesundheit und Integration des Landes Baden-Württemberg die Förderung von zunächst 19 Modellprojekten aus den Bereichen Tourismus, Kultur, Kinder- und Jugendarbeit sowie Sport. Ziel war es, unter kontrollierten Bedingungen eine schrittweise Öffnung von Betrieben verschiedenster gesellschaftlicher Bereiche in Baden-Württemberg zu ermöglichen. Auswahlkriterien für die Förderzusage war neben wissenschaftlicher Begleitung, einem ausführlichen Hygiene- und Testkonzept sowie einer stabilen 7‑Tages-Inzidenz unter 100 auch die Übertragbarkeit der Ergebnisse auf andere Bereiche des öffentlichen Lebens [27]. Als größter deutscher Freizeitpark bot sich der Europa-Park in Rust mit durchschnittlich 5,7 Mio. Eintritten pro Jahr als Modellstandort an, um einschlägige Erkenntnisse zu gewinnen, die auch für die Öffnung kleinerer Betriebe der Freizeit- und Tourismusbranche in Deutschland nützlich sind und ihnen eine möglichst schnelle Wiedereröffnung ermöglicht [37]. Ein weiterer Vorteil in der Auswahl des Europa-Park als Modellstandort besteht darin, dass hier sowohl innerbetriebliche Maßnahmen (das Personal betreffend) als auch außerbetriebliche Maßnahmen (die Gäste betreffend) untersucht werden können.

Inzwischen liegen für die im Rahmen der SARS-CoV-2-Pandemie implementierten Wiedereröffnungsstrategien erste länderspezifische Analysen vor [16]. Im Bereich der Unterhaltungs- und Event-Industrie existieren derzeit lediglich mathematische Vorhersagemodelle [2, 41] sowie die im Pandemieverlauf veröffentlichten Handlungsoptionen und Rahmenkonzepte mit Teilbereichen aus dem Infektions- und Arbeitsschutz [28]. Publizierte Rahmenkonzepte und Handlungsempfehlungen stützten sich weitestgehend auf fachlicher Expertise durch medizinisches Personal und berücksichtigen weder objektive Wirksamkeitskriterien (z. B. Direktmessungen in Form von Infektionsnachweisen) noch Indikatoren (z. B. Aerosolmessungen) oder die subjektive Wahrnehmung der Betroffenen (z. B. Gäste, Mitarbeitende; [1, 22, 28]). Tatsächlich verwendete Rahmenkonzepte, deren Evaluationsergebnisse und Daten über den gesamten Öffnungszeitraum unter Pandemiebedingungen sind bisher nicht veröffentlicht.

Ziel der Begleitforschung des Modellprojekts war es, die modellhafte Öffnung des Freizeitparks formativ und summativ zu evaluieren [13]. Schwerpunkte waren dabei neben der Anwendung des Hygienesystems des Europa-Park eine Analyse der vorgeschriebene SARS-CoV-2-Testungen (insbesondere unter Berücksichtigung der Sensitivität von SARS-CoV-2-Rapid-Antigen-Tests [RATs]; [6, 35]), begleitende Innenraum-Luftmessungen sowie eine umfassende Gästebefragung mit einem Fokus in der Bewertung der örtlichen Hygienemaßnahmen. Als Kriterien für eine gelungene Umsetzung des Modellvorhabens wurden dabei die Resonanz beim Publikum (subjektive Dimension) sowie die Auswertung von Aerosolmessungen und SARS-CoV-2-Testungen (objektive Dimension) gewählt.

## Methodik

Vor Beginn des Modellzeitraums wurde von der Geschäftsführung des Europa-Park und der Amtsleitung des Ministeriums für Soziales, Gesundheit und Integration Baden-Württemberg für die Abstimmung mit den örtlichen Behörden (Landratsamt bzw. Gesundheitsamt Ortenaukreis) ein Expertengremium für die Erarbeitung eines umfassenden Hygienesystems mit spezifischen Aufgaben aufgestellt:

1. Universitätsklinikum Freiburg, Stabsstelle Betriebsärztlicher Dienst
  - Begutachtung und Beratung
2. Palas GmbH (Karlsruhe)
  - Innenraumluftmessungen
  - Erarbeitung Leitfaden „Infektionsschutzgerechtes Lüften“
3. Fichtner*Piotrowsky Projektconsulting (Bayreuth)
  - Besuchermonitoring
  - Formative und summative Evaluation
4. Europa-Park Hygieneteam
  - Erarbeitung und Umsetzung von Hygiene- und Abstandsmaßnahmen

Das Hygienesystem des Europa-Park fußt dabei auf 4 Säulen:

1. Hygieneplan auf Basis von Handlungshilfen der Verwaltungs-Berufsgenossenschaft (VBG) sowie der Berufsgenossenschaft Nahrungsmittel und Gastgewerbe (BGN)
2. Hygienekonzepte für den Europa-Park und die Wasserwelt Rulantica
3. Checklisten zur betrieblichen Pandemieplanung des Bundesamtes für Bevölkerungsschutz und Katastrophenhilfe (BBK)
4. Arbeitsschutz und Gefährdungsbeurteilung gemäß SARS-CoV-2-Arbeitsschutzverordnung

## CIPP-Modell als Grundlage für das Modellvorhaben

**Figure 1:**
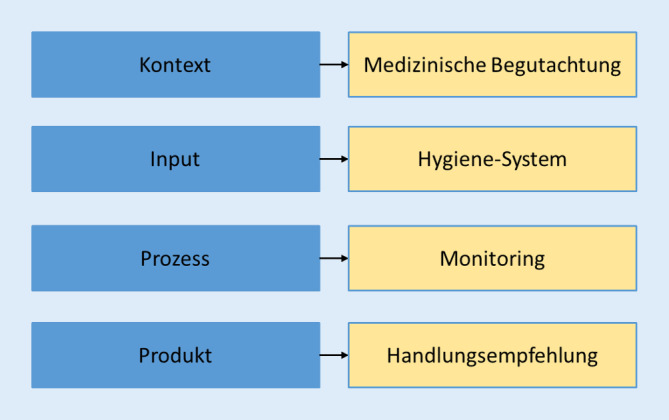

Das Modellvorhaben lässt sich, angelehnt an das CIPP-Modell nach Stufflebeam, in 4 Schwerpunktbereiche gliedern (Abb. 1; [38]).

Die *Kontextevaluation* dient der Identifikation der für die modellhafte Öffnung notwendigen Bedarfe und Rahmenbedingungen. In diesem Fall sind das die geltenden gesetzlichen Rahmenbedingungen in Deutschland bzw. Baden-Württemberg mit kontrollierten Besucherzahlen und Zugangsbeschränkungen sowie Hygieneauflagen.

Die *Inputanalyse* beschreibt die Planung der einzusetzenden Ressourcen (personell, finanziell und infrastrukturell) und Lösungsstrategien, die zur Umsetzung des Modellvorhabens notwendig sind. In diesem Zusammenhang ist das Hygiene- und Sicherheitskonzept seitens des ausgewählten Freizeitparks unter Einbezug von Expertenrunden mit interdisziplinären Teams aus den Bereichen Betriebswirtschaft, Sozialwissenschaft, Versorgungsforschung, Soziologie und Medizin entwickelt worden.

Die *Prozessanalyse* bildete das kontinuierliche Monitoring während der Projektlaufzeit. Hierzu wurden verschiedene Methoden genutzt:

1. Kontinuierliche Besucherbefragung zur Identifikation von Schwachstellen im Hygienekonzept
2. Follow-up-Besucherbefragung zur Identifikation von Infektionsketten in der Konsequenz des Besuchs
3. Aerosolmessungen mit wiederholten experimentellen Versuchen zur Bestimmung kritischer Räume
4. Teilnehmende (verdeckte) Beobachtung und Experteneinschätzung

Der Fokus der Prozessanalyse lag vor allem auf dem reibungslosen Ablauf des Modellvorhabens. Neben der Qualitätssicherung der Umsetzungsaktivitäten wurden potenzielle sowie tatsächlich aufgetretene Herausforderungen identifiziert und, sofern möglich, Lösungen etabliert. Hierzu erfolgten im Rahmen der formativen Evaluation kurzfristige Rückmeldungen mit Handlungsoptionen in Form von Dashboards an das verantwortliche leitende Personal im Europa-Park. Diese direkten Rückmeldungen waren zwingend erforderlich, da vor allem Zeitverzögerungen in der Umsetzung die Wirksamkeit von Maßnahmen zur Eindämmung der Pandemie erheblich beeinträchtigen können [29]. Prozessindikatoren sind z. B. die Akzeptanz der Maßnahmen bei den Parkgästen, die Einhaltung der AHA-L-Regeln während des Parkbesuchs sowie die Umsetzung des Hygienekonzepts. Diese Indikatoren wurden sowohl durch die qualitativen und quantitativen Hinweise in der Besucherbefragung, als auch durch die teilnehmende Beobachtung gemessen. In der Besucherbefragung wurde offen nach Orten und Gelegenheiten gefragt, an/bei denen sich die Gäste hinsichtlich des Infektionsrisikos bzw. des Verhaltens anderer unwohl fühlten. Diese qualitativen Nennungen wurden kodiert und quantitativ inhaltsanalytisch ausgewertet [17]. Es erfolgte eine zufällige Auswahl der identifizierten Orte für die teilnehmenden verdeckten Beobachtungen. Die Beobachtungen waren insofern verdeckt, weil das Evaluationsteam nicht als solches identifizierbar war, um Verhaltensmodifikation der Gäste durch die bloße Präsenz auszuschließen. Gegenstand der Beobachtung war das Verhalten der Gäste an kritischen Orten im Park, welches in Beobachtungsprotokollen festgehalten wurde. Die Befunde wurden innerhalb des Evaluationsteams unter Beteiligung des verantwortlichen Personals im Europa-Park diskutiert und Lösungsmöglichkeiten erörtert.

Die Ebene *Produkt* beinhaltet das Ergebnis des Modellvorhabens, also die abschließende Begutachtung des Projekts, resultierend in den Handlungsoptionen zu Öffnung und Betrieb von Freizeitdestinationen unter Pandemiebedingungen (vgl. Supplement 3).

## Rahmenbedingungen im Europa-Park

Mit einer Gesamtfläche von ca. 100 ha – hauptsächlich im Außenbereich – bietet der Europa-Park umfassend Platz, um auch bei erhöhtem Besuchsaufkommen einen Abstand von 1,5 m pro Gast zu gewährleisten. In der überwiegenden Zahl der Fälle erfolgt ein Besuch als Gruppe, mit entsprechend viel Platz zwischen den einzelnen Gruppen. Im Rahmen der Pandemie handelt es sich bei den Gruppen in der Mehrzahl um Familien (die in der Regel im selben Haushalt leben); organisierte Reisegruppen treten seltener auf (99 % der Befragten besuchten den Park alleine oder in einer Gruppe von maximal 10 Personen). Um die angeordneten räumlichen Distanzen zwischen Individuen respektive Gruppen zu gewährleisten, wurde im laufenden Parkbetrieb zusätzlich eine Besucherobergrenze definiert, die im vergangenen ersten Pandemiesommer 2020 in Abstimmung mit dem Gesundheitsamt des Ortenaukreises bei max. 50 % (20.000 Personen pro Tag) des regulären Besuchsaufkommens lag. Im Zuge der Modellöffnung 2021 startete der Europa-Park mit einer Limitierung auf rund 10.000 Besucher am Pfingstwochenende und erhöhte die Zutrittszahl im Verlauf der Projektphase auf bis zu 15.000 Gäste. Umfassende Vorkehrungen aus dem Jahr 2020 wurden fortgeführt und ergänzt (Auswahl):

- Online-Reservierung des Eintrittstickets (Möglichkeit der Kontaktnachverfolgung)
- Maskenpflicht (FFP2-Masken bzw. medizinischer Mund-Nasen-Schutz) in sensiblen Bereichen (z. B. Warteschlangen)
- Umfassendes Hygieneangebot mit z. B. über 300 Handwaschmöglichkeiten auf dem gesamten Parkgelände
- Engere Reinigungs- und Desinfektionsintervalle an den Attraktionen sowie Bereitstellung von zahlreichen Desinfektionsmittelspendern
- Warteschlangenmanagement (z. B. Errichtung von Acrylglas- und Kunststoffabtrennungen)
- Überwachung und Kontrolle durch das Personal an den Ein- und Ausgängen der Wartebereiche
- Lenkung der Besucherströme im Park durch visuelle Maßnahmen wie Abstandsmarkierungen und Leitmarker auf den Wegen
- Beschränkung des Zutritts auf Genesene (positives PCR-Testergebnis nach mindestens 28 Tagen und max. 6 Monaten), vollständig Geimpfte (Vorlage eines offiziellen Nachweises zur Beurkundung eines vollständigen Impfschutzes, 14 Tage nach vollständiger Impfung) und Getestete (negatives Ergebnis eines RAT oder PCR, das nicht älter als 24 h sein durfte) sowie eine rigorose Kontrolle der entsprechenden Nachweise
- Einrichtung einer eigenen Teststraße im Hauptparkplatz-Bereich und in der Folge die Trennung von vorab Getesteten und Ungetesteten
- Separates Testzentrum für Hotelbesucher

## Medizinische Begutachtung

Die medizinische Begutachtung erfolgte durch die Stabsstelle Betriebsärztlicher Dienst am Universitätsklinikum Freiburg. In allen Schwerpunktbereichen des Modellvorhabens erfolgten ein kontinuierlicher Austausch sowie eine fachliche Beratung einschließlich Bewertung des Hygienesystems des Europa-Park. Zusätzlich erfolgte eine initiale Berechnung der zu erwartenden positiven Testungen für die vor Ort an den zertifizierten Teststellen des Europa-Park durchgeführten SARS-CoV-2-Rapid-Antigen-Tests (RATs) bei unterschiedlichen Inzidenzen unter Berücksichtigung realistischer Sensitivitäten und Spezifitäten [5, 7]. Für die gesamte Parköffnungszeit (Start 20.05.2021) erfolgte ein Monitoring der SARS-CoV-2-RATs bis zur Parkschließung zum Saisonende am 9. Januar 2022. Für die Monate Mai 2021 mit einer durchschnittlichen Inzidenz von 61,1 sowie Januar 2022 mit einer Inzidenz von 264,4 (jeweils in Baden-Württemberg) wurde ein Vergleich zwischen zu erwartenden und tatsächlich dokumentierten positiven RATs vorgenommen.

## Innenraum-Luftmessungen und infektionsschutzgerechtes Lüften

Die Aerosolmessungen wurden durch die Firma Palas GmbH (Karlsruhe) durchgeführt. Ziel dieser Messungen war die Bestimmung des Risikos für eine Infektionsausbreitung über infektiöse Aerosole. Hierzu erfolgte ein experimenteller Versuchsaufbau in ausgewählten kritischen Innenräumen, sowohl im Bereich Gastronomie und Hotellerie als auch in diversen Warte- und Nutzungsräumen von Attraktionen. Die Messungen wurden über mehrere Tage bzw. Wochen durchgeführt, um repräsentative Ergebnisse für den normalen Parkbetrieb mit unterschiedlich hohen Gästezahlen zu erhalten. Zusätzlich zu den Messungen wurde die Besucheranzahl in den jeweiligen Räumen dokumentiert, um die Messdaten dazu ins Verhältnis setzen zu können. Im Laufe der Messungen wurde eine Attraktion mit kritischen Werten ausgewählt, um an zwei Besuchstagen beispielhaft die Auswirkung verschiedener Maßnahmen wie Frischluftzufuhr und Raumluftfilter zu testen und zu evaluieren. Aerosolanzahlkonzentration, Partikelgrößenverteilung, Feinstaubkonzentration (PM1, PM2,5, PM10), Konzentrationen von CO_2_ und tVOC („volatile organic compounts“) sowie Temperatur- und Feuchtemesswerte wurden nach dem Prinzip optischer Lichtstreuung am Einzelpartikel mithilfe von AQ Guard Geräten gemessen (https://www.palas.de/product/aq-guard). Weiterhin erfolgte eine Berechnung der Reproduktionszahl (auf Basis der Wels-Riley-Gleichung) zur Abschätzung des Infektionsrisikos über die gemessene Aerosolanzahlkonzentration und CO_2_-Konzentration [18, 34]. Im Ein- und Ausstiegsbereich des Fahrgeschäftes wurden 2 Luftfilter der Fa. Trotec (Heinsberg) mit je einem Luftdurchsatz von 1400 m^3^/h verwendet. Unter den räumlichen Gegebenheiten führt das zu einem Luftwechsel von 5 × Raumvolumen/h. Im Bahnverlauf wurden 2 Luftfilter der Firma Mann+Hummel (Ludwigsburg) mit einem Luftdurchsatz von 2500 m^3^/h und mit 850 m^3^/h verwendet. Unter den räumlichen Gegebenheiten führt das zu einem Luftwechsel von ca. 4–5 × Raumvolumen/h. Am ersten Versuchstag kamen die Luftfilter von 5:00 Uhr bis 13:00 Uhr zum Einsatz, am zweiten von 13:00 bis 22:00 Uhr. Unter Berücksichtigung der Messergebnisse sowie aktueller Empfehlungen zu Raumlüftungen wurde ein Ablaufdiagramm für notwendige Risikobeurteilungen der Räumlichkeiten abgeleitet [19, 23, 39].

## Gästebefragung

Die Überprüfung der umgesetzten Maßnahmen aus Sicht des Publikums erfolgte durch ein kontinuierliches Besuchermonitoring mit Follow-up-Befragung. Zum ersten Messzeitpunkt (t1) wurden in den ersten beiden Öffnungswochen nach dem zweiten Lockdown (21.05.2021 bis 03.06.2021) Gäste am Haupt- und Hotelausgang des Parks mündlich befragt. Die zweite Messung (t2) schloss sich jeweils mindestens 14 Tage nach dem im Rahmen von t1 durchgeführten Interview an. Diese Zeitdifferenz wurde in Abstimmung mit dem Gesundheitsamt des Ortenaukreises sowie der Stabsstelle Betriebsärztlicher Dienst am Universitätsklinikum Freiburg festgelegt und richtete sich nach der zu erwartenden Inkubationszeit der COVID-19-Erkrankung. Berücksichtigt wurde dabei der sich verändernde Infektionseinfluss der verschiedenen SARS-CoV-2-Varianten hinsichtlich Ansteckungsgrad und Inkubationszeitraum. Schwerpunkte der Befragungen waren Basisdaten (z. B. Anreise, Herkunftsland), Aufenthalt (z. B. Dauer, Anzahl der Besuche, Übernachtungen), Zufriedenheit (unterschiedliche Kontexte), Bedingungen für Zugangsberechtigung (3G) und Bewertung der Hygienemaßnahmen (z. B. kritische Orte, Verbesserungsvorschläge).

Im Rahmen der t1-Messung erfolgte die Datenerfassung zunächst mittels computergestützter Face-to-Face-Interviews (CAPI) durch geschultes, mehrsprachiges Personal mit Parkgästen als kontinuierliches Besuchermonitoring. Nach Abschluss dieses Interviews wurden die Besucher gebeten, ihre Mailadresse für eine erneute Kontaktaufnahme (t2) nach Ablauf von 14 Tagen anzugeben. Zur t2-Messung wurde ein Onlinesurvey eingesetzt, um weitere Daten zum Infektionsrisiko zu sammeln. Dabei wurde ein Token generiert, sodass nur eine einmalige Teilnahme möglich war.

Die Auswahl der Befragten vor Ort zum Zeitpunkt t1 erfolgte zufallsbasiert. Durch die Aufnahme einiger Basisdaten konnte die so gezogene Stichprobe nach Abschluss des Modellprojekts mit parkinternen Daten abgeglichen werden, wozu u. a. ausgewählte Strukturmerkmale der Gäste gehörten. Dies diente der Abschätzung der Repräsentativität der Stichprobe.

Um die Ausfälle zwischen den beiden Messzeitpunkten möglichst gering zu halten (Lost-to-Follow-up), wurden Anreize („incentives“) in Form von Gutscheincodes am Ende des Interviews t1 angekündigt. Anschließend wurden diese nach Teilnahme bei t2 und erfolgreicher Übermittlung des Onlinefragebogens ausgegeben. Mehrfache Erinnerungs-E-Mails an säumige Probanden dienten der Erhöhung der Rücklaufquote. Die Rekrutierungsphase für die t2-Befragung war mit dem 05.07.2021 abgeschlossen. Die qualitativen Informationen wurden kodiert und inhaltsanalytisch ausgewertet. Die quantitativen Daten wurden weitestgehend deskriptiv ausgewertet. Gruppenunterschiede wurden mittels t‑Tests und Mann-Whitney-U-Tests bestimmt und Korrelationen wurden mittels Kontingenzanalyse sowie Korrelationsanalyse nach Bravais-Pearson berechnet. Angesichts des kurzen Planungshorizonts wurde kein Prätest des Fragebogens durchgeführt, es wurde jedoch auf bewährte Fragen aus dem jährlichen Besuchermonitoring zurückgegriffen.

## Ergebnisse

### SARS-CoV-2-Rapid-Antigen-Testungen

Während der Parköffnung von Mai 2021 bis Januar 2022 wurden an allen Teststationen insgesamt 133.081 RATs durchgeführt, wobei die monatliche Inanspruchnahme abhängig war vom Besuchsaufkommen und den Vorgaben der jeweils gültigen Corona-Verordnungen (Tab. 1). Abhängig von der jeweiligen Inzidenz sowie der vorherrschenden Virusvariante (Beta, Delta oder Omikron) ergaben sich maximale Positivraten von 0 % (Beta), 0,14 % (Delta) und 0,21 % (Omikron). Im Mai 2021 lag die zu erwartende Rate an positiven Befunden bei null, ausgehend von einer angenommenen Sensitivität von 71,2 % oder 57,1 % der eingesetzten RATs (vgl. Supplement 1), was mit der der tatsächlich aufgetretenen Rate übereinstimmte. Im Zeitraum Januar 2022 wurden insgesamt 18 positive Befunde erhoben, dies entspricht den bei einer durchschnittlichen Sensitivität von 71,2 % sowie Spezifität von 98,9 % zu erwartenden 17 positiven Befunden bei 8494 Tests. Die Auswertung der ungültigen Testungen (negative Positiv-Kontrolle) ergab eine durchschnittliche Rate von 0,31 %.

**Table 1:** 

Monat	Tests gesamt (*n*)	Tests negativ (*n*)	Tests negativ (%)	Tests positiv (*n*)	Tests positiv (%)	Tests neutral (*n*)	Tests neutral (%)
Mai 2021	21.276	21.208	99,68	0	0,00	68	0,32
Juni 2021	46.739	46.556	99,61	2	0,00	181	0,39
Juli 2021	1465	1461	99,73	0	0,00	4	0,27
August 2021	15.802	15.738	99,59	0	0,00	64	0,41
September 2021	12.637	12.602	99,72	2	0,02	33	0,26
Oktober 2021	8936	8904	99,64	3	0,03	29	0,32
November 2021	3461	3445	99,54	5	0,14	11	0,32
Dezember 2021	14.271	14.246	99,82	7	0,05	18	0,13
Januar 2022	8494	8466	99,67	18	0,21	10	0,12
*Gesamt*	*133.081*	*132.626*	*99,66*	*37*	*0,03*	*418*	*0,31*

### Innenraum-Luftmessungen

**Figure 2:**
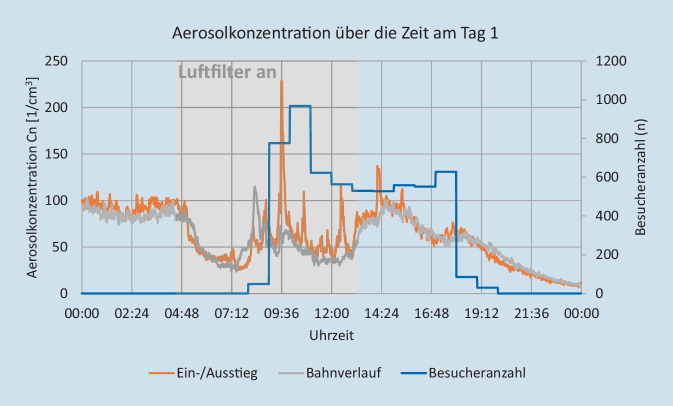

In Abb. 2 und 3 sind die Aerosolkonzentrationen der beiden Tage für den Ein- bzw. Ausstieg und den Bahnverlauf der ausgewählten Attraktion zu sehen. Der Verlauf der Aerosolkonzentration an den beiden Messstellen ist im Mittel ähnlich. Das Aerosol scheint sich relativ gleichmäßig in der Bahn zu verteilen. Im Ein‑/Ausstiegsbereich zeigt die Aerosolkonzentration stärkere Variation. Das ist auf die Bewegungsaktivität der Besucher zurückzuführen, die anders als bei der sitzenden Teilnahme an der Fahrt weniger eingeschränkt ist. Hier ist das „Aerosol-Grundrauschen“ deutlich höher, z. B. durch kleine Partikel, die vom Boden wieder aufgewirbelt werden oder sich von der Kleidung, z. B. beim Aufstehen ablösen. An beiden Tagen reduzieren die Luftfilter die Aerosolkonzentration gegenüber der Zeit mit ausgeschaltetem Luftfilter deutlich um durchschnittlich 46 % im Ein‑/Ausstiegsbereich und 43 % im Bahnverlauf (Tab. 2; Abb. 4 und 5). Basierend auf den Messergebnissen wurde eine verbindliche Entscheidungsvorlage zur Anwendung einer Risikobeurteilung für infektionsschutzgerechtes Lüften erstellt (Supplement 2).

**Figure 3:**
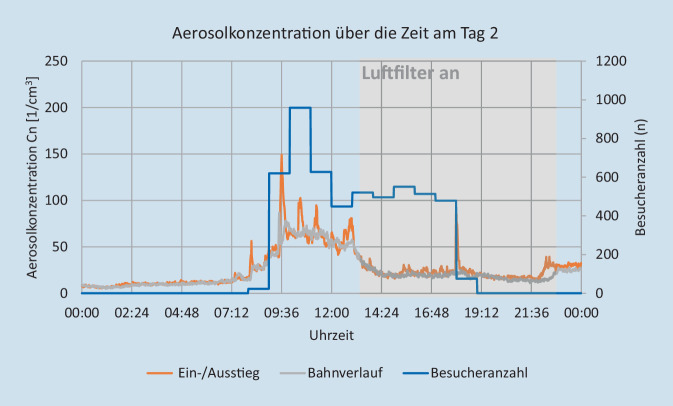

**Table 2:** 

Tag	Betrachtete Zeiträume		Ein‑/Ausstieg	Bahnverlauf
	Mit ausgeschaltetem Luftfilter	Mit angeschaltetem Luftfilter		
1	04:30–05:00 Uhr	05:30–06:00 Uhr	Reduktion um 53 %	Reduktion um 41 %
1	13:30–14:00 Uhr	12:30–13:00 Uhr	Reduktion um 30 %	Reduktion um 48 %
2	12:30–13:00 Uhr	13:30–14:00 Uhr	Reduktion um 56 %	Reduktion um 45 %
2	22:30–23:00 Uhr	21:30–22:00 Uhr	Reduktion um 45 %	Reduktion um 39 %
*Mittelwert*			*Reduktion um 46* *%*	*Reduktion um 43* *%*

**Figure 4:**
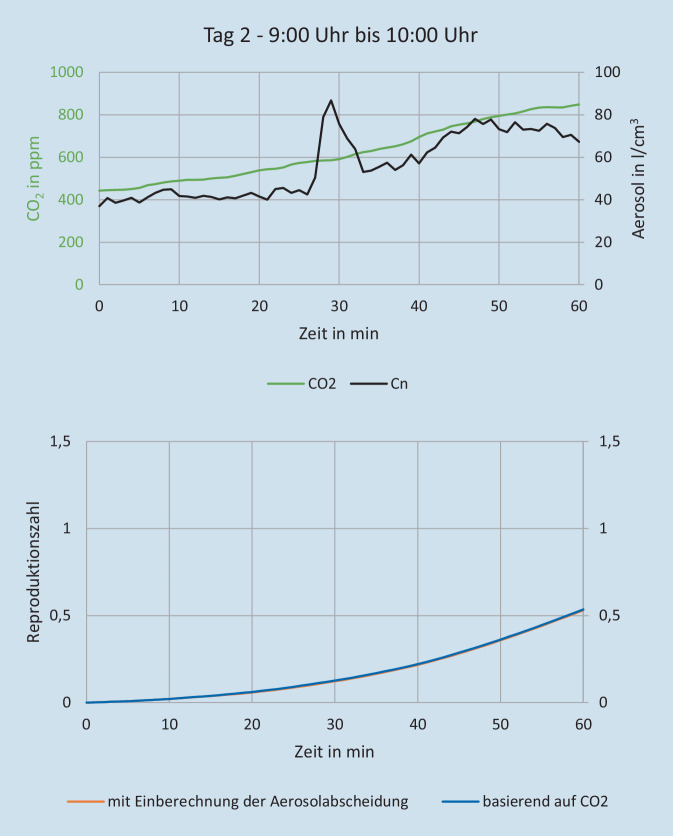

**Figure 5:**
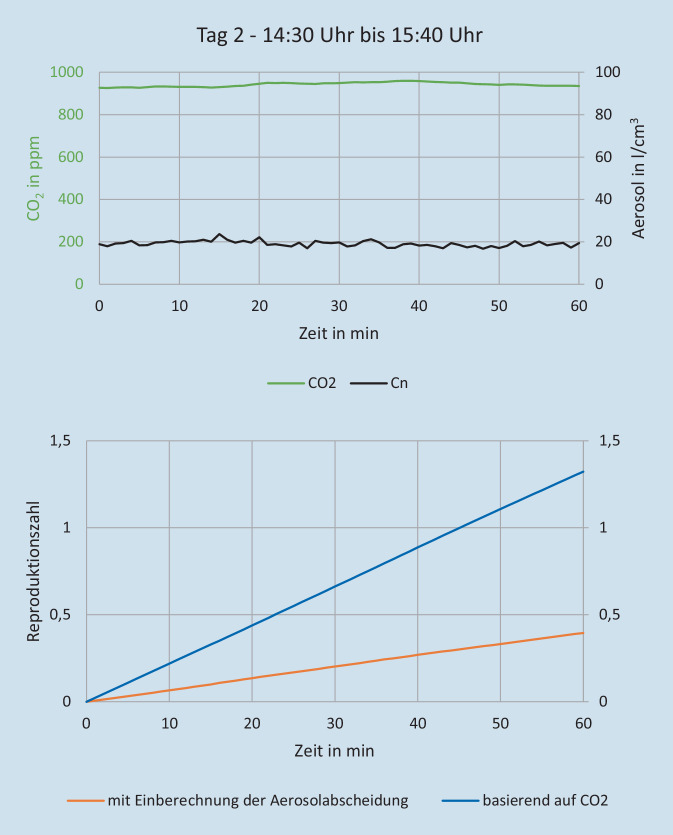

### Gästebefragung

Insgesamt wurden 1425 gültige Interviews zu t1 geführt. 1131 Personen willigten in die erneute Kontaktaufnahme für die Messung zu t2 ein. 695 Personen riefen den nach Ablauf von 14 Tagen verschickten Survey-Link auf mit insgesamt 664 gültigen Online-Antworten während der Projektlaufzeit (Gesamtdaten). Damit ergibt sich eine Rücklaufquote für t2 von 59 %.

#### Kritische Orte

Um während der Testöffnung kritische Bereiche aufzudecken und damit die Möglichkeit einer Nachbesserung zu schaffen, wurden die Teilnehmer der Studie zu t1 gefragt, ob Orte oder Gelegenheiten im Park selbst oder im Rahmen ihres Aufenthalts außerhalb im Umfeld des Parks wahrgenommen wurden, an oder bei denen sie sich hinsichtlich einer möglichen Ansteckungsgefahr unwohl fühlten. Insgesamt verneinte zu jedem Zeitpunkt eine deutliche Mehrheit diese beiden Fragen. Bis zu 29 % der Befragten nannten im Laufe der t1-Phase kritische Orte oder Gelegenheiten im Park, maximal 5 % Orte außerhalb der Anlage. Am häufigsten wurden Warteschlangen im Allgemeinen als kritischer Ort genannt. Auch der Eingangsbereich wurde als sehr dichte Zone empfunden. Dies war vor allem zu den Stoßzeiten am Morgen beim Einlass und am Abend bei Parkschluss ein relevanter Faktor. Unwohl hinsichtlich Ansteckung fühlten sich manche Gäste auch auf den Toiletten. Hier wurde vor allem bemängelt, dass Desinfektionsspender fehlten und sich vereinzelt zu viele Personen in den Waschräumen aufhielten. Als weitere kritische Areale wurden Außenbereiche und Wege genannt, an denen hohe Besucherdichten auftreten. Dies sind vor allem Hauptwege und Plätze vor beliebten Attraktionen auf denen sich große Besucherströme bewegen. Fehlende Rücksichtnahme anderer Gäste (falsches oder ausbleibendes Tragen der Mund-Nasen-Bedeckung, Rauchen, fehlende Distanzwahrung) wurde dabei am häufigsten bemängelt.

#### Verbesserungsvorschläge

Im Onlinesurvey der t2-Messung konnten Teilnehmer, die nicht vollständig zufrieden mit der Eindämmung des Risikos einer möglichen Ansteckungsgefahr anlässlich ihres zurückliegenden Parkbesuchs waren, im Nachhinein Verbesserungsvorschläge zur Erhöhung der Sicherheit im Park abgeben. Neben vielen Einzelnennungen wurden vor allem stärkere und striktere Kontrolle durch das Personal vorgeschlagen. Dies solle konsequent die Wartebereiche der Attraktionen überwachen und deviante Personen auf die geltenden Abstands- und Maskenvorschriften hinweisen. Durch regulierende Maßnahmen wie Einbahnstraßen und Markierungen, aber auch durch Kontrollpersonal können enge Plätze und Wege im Park entzerrt werden. Ebenso könnte aus Sicht der Probanden eine Erhöhung der Kapazität in der Gastronomie zu kürzeren unkontrollierten Wartezeiten führen. Insgesamt wünschten sich viele Gäste mehr Desinfektionsmöglichkeiten, vor allem am Ausgang der Attraktionen und in Toiletten, sowie mehr Abstandsmarkierungen. Bereits in den t1-Interviews wurde deutlich, dass vor allem rücksichtsloses Verhalten anderer Parkgäste als persönliches Sicherheitsrisiko wahrgenommen wurde. Nach Ansicht der betroffenen Besucher könnte hierbei neben intensiveren Kontrollen durch Parkpersonal möglicherweise eine niedrigere Besucherobergrenze im Park insgesamt Abhilfe schaffen.

### Handlungsoptionen

Die Erkenntnisse, welche im Rahmen der Datenerhebung während des Modellprojekts gewonnen werden konnten, wurden gebündelt und auf ihrer Basis Handlungsoptionen für die sichere Öffnung und den Betrieb von Freizeitparks unter Pandemiebedingungen formuliert (Supplement 3). Wesentliche Elemente beinhalten unter Infektions- und Arbeitsschutzgesichtspunkten die nachfolgenden Teilbereiche:

1. Gefährdungsbeurteilung des Standortes
2. Betrieblicher Pandemieplan zum Arbeitsschutz
3. Strategie zum Umgang mit Verdachtsfällen
4. Arbeitsmedizinische Vorsorge und Schutz besonders gefährdeter Personen
5. Schutzmaßnahmen für Flächen, Bereiche und Einrichtungen, die von Gästen aufgesucht werden
6. Abstimmung mit dem örtlichen Gesundheitsamt
7. Evaluation der getroffenen Maßnahmen

Unter kontinuierlicher Anwendung der Handlungsoptionen konnte der Europa-Park während der gesamten Saison von Mai 2021 bis Januar 2022 geöffnet bleiben.

## Diskussion

Im Rahmen des Modellprojekts ist es nicht nur gelungen, eine initiale Öffnung des Freizeitparks zu realisieren, es konnten auch wertvolle Handlungsoptionen für den zukünftigen Betrieb unter Pandemiebedingungen erarbeitet werden. Die abgeleiteten Handlungsoptionen wurden mittels interner Dashboards kontinuierlich durch zeitnahe und direkte Rückmeldungen formativ evaluiert, wobei der Schwerpunkt auf der Anwendung und Optimierung des Hygienesystems des Europa-Park lag. Umfassende Daten konnten zu den vorgeschriebenen SARS-CoV-2-Testungen erhoben werden, begleitende Innenraum-Luftmessungen ermöglichten die Etablierung einer Risikobeurteilung für infektionsschutzgerechtes Lüften, und eine umfassende Gästebefragung mit einem Schwerpunkt in der Bewertung der örtlichen Hygienemaßnahmen führte zur Maßnahmenoptimierung während der gesamten Öffnungsphase. Die vorliegenden Daten und Handlungsoptionen können politischen Entscheidungsträgern und Betreibern anderer, vergleichbarer Freizeiteinrichtung als Orientierung dienen, um auch auf mögliche zukünftige Pandemiesituationen angemessen reagieren zu können. Basierend auf den im Vorfeld verfügbaren internationalen Vorschlägen bilden die Ergebnisse unserer Studie, unter Berücksichtigung der gesetzlichen Infektions- und Arbeitsschutzvorgaben, die Grundlage für die abgeleiteten Handlungsoptionen. Diese muss bei kleineren Einrichtungen entsprechend den vor Ort zur Verfügung stehenden Ressourcen sowie den spezifischen Vorgaben der örtlichen Gesundheitsbehörden entsprechen angepasst und auf Kernelemente reduziert werden (z. B. Testen, Lüften, Abstand und Masken).

Zur Eindämmung der SARS-CoV-2-Pandemie hat der Gesetzgeber kontinuierlich auf Bundes- und Landesebene eine Vielzahl von Maßnahmen durch Gesetze und Verordnungen umgesetzt, um SARS-CoV-2-Infektionen innerhalb der Bevölkerung effektiv einzudämmen und COVID-19-Erkrankungen zu verhindern. Schwerpunkte waren und sind hierbei für die Bevölkerung, aber auch innerhalb der betrieblichen Pandemieplanung, die AHA+L + A-Formel sowie eine umfassende Test- und Impfstrategie. Zu Beginn der Jahres 2021 musste teilweise immer noch auf Erfahrungen aus der Influenza-Pandemie von 1918 zurückgegriffen werden [25, 36]. Es zeigt sich zunehmend im Rahmen der SARS-CoV-2-Pandemie, dass die Anwendung der AHA+L + A-Formel [8, 14, 31], insbesondere das Tragen von Masken sowie Abstand [3, 33, 40] und die Etablierung umfassender SARS-CoV-2-Impfprogramme [9] wirksame Interventionen darstellen. Die breite Anwendung von SARS-CoV-2-RATs bei asymptomatischen Bevölkerungsteilen wird teilweise kontrovers diskutiert [26]. SARS-CoV-2-Rapid-Antigen-Testungen bei potenziell asymptomatischen Probanden sollten immer unter Berücksichtigung der Sensitivität und möglicher falsch-positiver Testergebnisse erfolgen [11, 20]. Auffallend bei den am Europa-Park durchgeführten RATs ist die grundsätzlich sehr niedrige Rate an positiven Befunden. Dies könnte an einer Selektion der Grundgesamtheit der Parkbesucher liegen, da symptomatische Personen wahrscheinlich unterrepräsentiert sind. Bei niedriger Viruslast und unzureichender Probenentnahme ist die Aussagekraft von RATs zusätzlich eingeschränkt und führt möglicherweise zu einer falsch-niedrigen Rate an positiven Testergebnissen [6, 35]. Insbesondere bei kombinierten Hotel- und Parkbesuchen wurden die Gäste intensiv im Vorfeld über die geltenden Impf- und Testvoraussetzungen informiert, einschließlich Testungsempfehlung vor Anreise, so dass die durchgehend niedrigen Raten positiver Testungen auf die erfolgreiche Kommunikation mit und Berücksichtigung der Empfehlungen durch die Gäste zu sehen ist. Umfassende Teststrategien im Umfeld von Freizeitdestinationen sollten daher von einer entsprechenden Kommunikationsstrategie begleitet werden.

Als Hauptübertragungsweg von SARS-CoV‑2 ist die Atemluft anzusehen [42]. Damit auch in Innenräumen ein Infektionsrisiko durch SARS-CoV‑2 effektiv reduziert werden kann, werden Raumluftwechselraten von 6 pro Stunde empfohlen [23]. Der Austausch der Luft kann durch moderne raumlufttechnische Anlagen (RLT), Spaltlüftung, Stoßlüftung, Fensterlüftung dauerhaft geöffnet oder Querlüftung erreicht werden. Die Raumluft kann ergänzend durch umluftbetriebene Raumluftreiniger gereinigt werden [18]. Unter Berücksichtigung der jeweils vorhandenen Gebäudetechnik und Beanspruchung, wie raumlufttechnische Anlagen, Fensterlüftung, max. Personenanzahl sowie umfassende Raumluftmessungen, können die Innenbereiche in kritisch oder unkritisch unterteilt werden, eine entsprechende Entscheidungsvorlage zur Anwendung einer Risikobeurteilung wird von den Autoren vorgeschlagen (vgl. Supplement 2). Insbesondere durch den Einsatz von Raumluftreinigern konnte in kritischen Innenräumen eine Reduktion der Aerosolkonzentration mit einer daraus anzunehmenden Senkung des Infektionsrisikos erzielt werden und somit vorherige Untersuchungen in schulischen Unterrichtsräumen bestätigt werden [18].

Elementarer Bestandteil einer erfolgreichen Überwindung der SARS-CoV-2-Pandemie ist eine hohe Maßnahmen-Compliance innerhalb der Bevölkerung [4]. Insbesondere das Tragen von Masken sowie Abstand spielen dabei eine kritische Rolle [24, 33]. Unsere Ergebnisse zeigen, dass ein konsequentes Besuchermonitoring ein entscheidendes Instrument zur Überprüfung der Akzeptanz der Maßnahmen auf Seiten des Publikums ist. Neben diesem Aspekt können Abweichungen in der Umsetzung der Hygienekonzepte identifiziert und lokalisiert werden. Damit lassen sich Veränderungen in der Publikumsmeinung, die beispielsweise durch verschiedenen Pandemiephasen bedingt sein können, abbilden, so dass schnellstmöglich bestehende Hygienekonzepte angepasst und weitere Regelungen getroffen werden können, um ein hohes Niveau an Akzeptanz zu erzielen. Aufgrund der medialen Berichterstattung, einschließlich Inzidenzen der jeweilig vorherrschenden SARS-CoV-2-Variante, können sich die Maßnahmen-Compliance und die subjektive Wahrnehmung der Besucher ändern. Ein erhöhtes Sicherheitsbedürfnis und veränderte Wahrnehmung kritischer Bereiche kann die Folge sein, was eine kontinuierliche effektive Anpassung der Hygienekonzepte erfordert. Exemplarisch sind hier als kritischer Bereich Wartezonen in der Gastronomie zu nennen, bei denen der Personaleinsatz in der Regel unter betriebswirtschaftlichen, und weniger unter pandemischen Gesichtspunkten reguliert wird. Dabei erfolgte die Anpassung der Maßnahmen teilweise aufgrund subjektiv wahrgenommener Risiko- und Sicherheitsbedürfnisse und nicht anhand einer unabhängigen Risikoanalyse, um insbesondere auch einer möglichen Diskrepanz zwischen wahrgenommenem und tatsächlichen Risiko gerecht zu werden [30].

Limitierend bei diesem Modellprojekt ist, dass teilweise kritische Unternehmensdaten, z. B. Besucherströme innerhalb der Freizeitdestination sowie relevante persönliche Daten, z. B. SARS-CoV-2-Infektionen, nicht berücksichtigt werden konnten, welche u. a. für zukünftige vorausschauende Simulationen bzw. weitere Pandemieplanung hilfreich wären [2, 12].

Dennoch zeigt dieses Modellprojekt erstmalig die konkrete Umsetzung der bisher vorgeschlagenen Handlungsoptionen und Rahmenkonzepte unter Berücksichtigung der gesetzlichen Arbeits- und Infektionsschutzvorgaben mit dauerhafter Öffnung über den gesamten Beobachtungszeitraum ohne notwendige Beschränkungen durch die örtlichen Gesundheitsbehörden aufgrund deren Kenntnisse zum lokalen bzw. überörtlichen Infektionsgeschehen im Zusammenhang mit den Parkbesuchen.

Zusammenfassend konnte durch die Triangulation von verschiedenen Datenquellen eine umfassende und multidisziplinäre Begutachtung des Modellprojekts aus verschiedenen Perspektiven erfolgen. Durch die Zusammenführung verschiedener Datenquellen und Forschungsmethoden konnten die Mängel und Schwächen einzelner Forschungsansätze (z. B. Subjektivität von Gästebefragungen, Repräsentativität von qualitativen Daten, Systematik und Fairness verdeckter Beobachtungen, Übertragbarkeit von Labormessungen) ausgeglichen und die Aussagekraft der Ergebnisse bestärkt werden [15]. Die so gewonnenen Erkenntnisse liefern konkrete Handlungsoptionen für politische Entscheidungsträger und für Betreiber von Freizeiteinrichtungen zur sicheren Öffnung ihrer Anlagen unter Pandemiebedingungen.

## Supplementary Information






