# Supplementary material for: Development of specific guidance for the safe opening and operation of recreational destinations under pandemic conditions
Source: Zentralbl Arbeitsmed Arbeitsschutz Ergon. 2022 Oct 7;72(6):267–77. [Article in German] doi: 10.1007/s40664-022-00480-y (PMC9540292; doi:10.1007/s40664-022-00480-y)
Supplement: Supplementary file 1 [file 40664_2022_480_MOESM1_ESM.docx]

**Supplement 1:** **Tabellen zur Abschätzung positiver SARS-CoV-2 Rapid-Antigen-Teste**

**Tabelle S1**: Abschätzung positiver SARS-CoV-2 Rapid-Antigen-Teste bei einer angenommenen idealen Sensitivität von 95% sowie Spezifität von 99,9% (<https://diagnostics-global-health.github.io/rechner/>)

| Inzidenz | positiv (n) | **negativ (n)** | falsch positiv (n) | falsch negativ (n) | positiv prädiktiver Wert (%) | negativ prädiktiver Wert (%) |
| --- | --- | --- | --- | --- | --- | --- |
| 500^a^ | 5 | 994 | 1 | 0 | 82,68 | 99,97 |
| 264,4 | 3 | 996 | 1 | 0 | 71,58 | 99,99 |
| 200 | 2 | 997 | 1 | 0 | 65,56 | 99,99 |
| 100 | 1 | 998 | 1 | 0 | 48,74 | 99,99 |
| 61,1 | 1 | 998 | 1 | 0 | 37,74 | 100 |
| 50 | 0 | 999 | 1 | 0 | 32,21 | 100 |

**Tabelle S2:** Abschätzung positiver SARS-CoV-2 Rapid-Antigen-Teste bei einer Sensitivität von 71,2% sowie Spezifität von 98,9% [7]

| Inzidenz | positiv (n) | **negativ (n)** | falsch positiv (n) | falsch negativ (n) | positiv prädiktiver Wert (%) | negativ prädiktiver Wert (%) |
| --- | --- | --- | --- | --- | --- | --- |
| 500 | 4 | 984 | 11 | 1 | 24,54 | 99,85 |
| 264,4 | 2 | 986 | 11 | 1 | 14,65 | 99,92 |
| 200 | 1 | 987 | 11 | 0 | 11,48 | 99,94 |
| 100 | 1 | 988 | 11 | 0 | 6,08 | 99,97 |
| 61,1 | 0 | 988 | 11 | 0 | 3,81 | 99,98 |
| 50 | 0 | 989 | 11 | 0 | 3,14 | 99,99 |

**Tabelle S3:** Abschätzung positiver SARS-CoV-2 Rapid-Antigen-Teste bei einer Sensitivität von 57,1% sowie Spezifität von 99,3% [5]

| Inzidenz | positiv (n) | **negativ (n)** | falsch positiv (n) | falsch negativ (n) | positiv prädiktiver Wert (%) | negativ prädiktiver Wert (%) |
| --- | --- | --- | --- | --- | --- | --- |
| 500 | 3 | 988 | 7 | 2 | 29,07 | 99,78 |
| 264,4 | 2 | 990 | 7 | 1 | 17,78 | 99,89 |
| 200 | 1 | 991 | 7 | 1 | 14,05 | 99,91 |
| 100 | 1 | 992 | 7 | 0 | 7,55 | 99,96 |
| 61,1 | 0 | 992 | 7 | 0 | 4,75 | 99,97 |
| 50 | 0 | 993 | 7 | 0 | 3,92 | 99,98 |
