# Supplementary material for: Development of specific guidance for the safe opening and operation of recreational destinations under pandemic conditions
Source: Zentralbl Arbeitsmed Arbeitsschutz Ergon. 2022 Oct 7;72(6):267–77. [Article in German] doi: 10.1007/s40664-022-00480-y (PMC9540292; doi:10.1007/s40664-022-00480-y)
Supplement: Supplementary file 2 [file 40664_2022_480_MOESM2_ESM.docx]

Supplement 2: Entscheidungsvorlage zur Anwendung einer Risikobeurteilung im Rahmen eines infektionsschutzgerechten Lüftens

Das nachfolgende Ablaufdiagramm dient der eventuell notwendigen Risikobeurteilung der Räumlichkeiten. Das Risiko wird mithilfe der Erkenntnisse aus den Messungen einer exemplarischen Fahrattraktion, sowieso den wissenschaftlichen und medizinischen Kenntnissen beurteilt.

Erläuterung zu den Maßnahmen:

^1^Entweder maximale Personenanzahl begrenzen um den Ausstoß möglicher kontaminierter Partikel zu verringern oder die Aufenthaltsdauer begrenzen um die Expositionszeit zu verkürzen.

^2^Raumluftreiniger werden je nach Raumgröße oder maximaler Personenanzahl berechnet und ausgewählt.

^3^Frischluftzufuhr durch raumlufttechnische Anlage, mobile Lüftung gewährleisten.
