# Supplementary material for: Development of specific guidance for the safe opening and operation of recreational destinations under pandemic conditions
Source: Zentralbl Arbeitsmed Arbeitsschutz Ergon. 2022 Oct 7;72(6):267–77. [Article in German] doi: 10.1007/s40664-022-00480-y (PMC9540292; doi:10.1007/s40664-022-00480-y)
Supplement: Supplementary file 3 [file 40664_2022_480_MOESM3_ESM.docx]

Supplement 3: Handlungsoptionen für die sichere Öffnung und den Betrieb von Freizeitparks unter Pandemiebedingungen

Die hier formulierten Handlungsoptionen dienen nicht nur als Hilfestellung für eine sichere Öffnung von Freizeit- und Tourismusdestinationen, sie fassen darüber hinaus die Parameter zusammen, die für den nachhaltigen Betrieb dieser Anlagen unter Pandemiebedingungen notwendig sind. Für die internationale Nutzung wurde sie auch auf Englisch übersetzt.

1. Gefährdungsbeurteilung des Standortes

Im Rahmen der Pandemiesituation sollte eine systematische Gefährdungsbeurteilung aller relevanten Bereiche hinsichtlich Ansteckungsrisiko durchgeführt werden.

Relevante Gefahrenräume:

- Indoor-Bereiche:

- Warte- und Ausgangsbereiche

- Gastronomie / Restaurants

- Sanitäre Anlagen

- Shops

- Indoor-Attraktionen und Shows

- Outdoor-Bereiche:

- Wege mit hohem Personenaufkommen, z.B. zentrale Plätze, Kreuzungen, Ein- und Ausgänge

- Betriebsinterne Bereiche:

- Shops

- Büros

- Servicebereiche

- Kantinen und Pausenräume

2. Entwicklung eines betrieblichen Pandemieplans zum Arbeitsschutz

Die zusätzlich erforderlichen Maßnahmen des betrieblichen Infektionsschutzes sollten zentral in einem betrieblichen Hygienekonzept festgehalten und unter Einsatz einer Task Force systematisch umgesetzt werden. Allgemein gilt: alle Maßnahmen müssen umfassend kommuniziert und entsprechende Unterweisungen standardisiert werden. Bei internationalem Personal ist auf die Mehrsprachigkeit des Informationsmaterials zu achten, um die Barrierefreiheit zu gewährleisten

Mögliche innerbetriebliche Maßnahmen:

- Gewährleistung zur Einhaltung des Sicherheitsabstandes unter den Mitarbeitenden
- Berührungen vermeiden (durch Handlungsanweisungen)
- Hust-, Nies- und Hygieneetikette etablieren und entsprechend kommunizieren
- Sanitäre Infrastruktur schaffen (Wasser, Seife, Desinfektionsmöglichkeiten, Einmalhandtücher)
- Mund-Nasen-Schutz (MNS) sowie FFP2-Masken für Mitarbeitende zur Verfügung stellen
- Anweisungen zum korrekten Tragen von MNS/FFP2 formulieren (in Wort und Bild)
- Lüftungskonzept etablieren und sofern erforderlich Raumluftreiniger aufstellen
- Wartungszyklen für Belüftungsanlagen sicherstellen
- Den Betrieb raumlufttechnischer Anlagen ohne Filtration vermeiden
- Mehrfachbelegung von Räumen organisieren und ggf. Raumbelegung definieren
- Belegschaft in definierte Arbeitsgruppen gliedern, um innerbetriebliche Kontakte zu reduzieren und um im Falle einer Infektion innerhalb der Gruppe die von Quarantäne betroffene Anzahl an Personen gering zu halten
- Sofern umsetzbar: Home-Office ermöglichen
- Betriebsbedingte Kontakte in Räumlichkeiten mit hoher Personendichte (Kantine, Aufzüge, Flure) reduzieren
- Sofern trotz betrieblicher Maßnahmen Personenkontakt nicht vermeidbar ist, Hinweisschilder und Markierungen anbringen
- Personenzahlen pro Räumlichkeit reduzieren (Obergrenze 10 Quadratmeter pro Person)
- In Bereichen, in denen der Mindestabstand von 1,5m nicht eingehalten werden können, sollten mechanische Barrieren (z.B. durch Acrylglas) geschaffen werden (z.B. an Ausgaben, Kassen, Help Desks)
- In Bereichen, in denen Warteschlangen entstehen können (z.B. Kantine) Abstandsmarkierungen anbringen
- Maßnahmenplan für den Umgang mit Kunden und im Außendienst etablieren
- Dienstreisen reduzieren und wenn möglich Online- und Telefonkonferenztools einsetzen
- Arbeitsmittel und Werkzeuge personenbezogen einsetzen und Reinigungsintervalle erhöhen
- Arbeitszeitmanagement (ggf. Schichtbetrieb einführen zur Verringerung innerbetrieblicher Personenkontakte)
- Kontaktdaten und Kontaktzeit betriebsfremder Personen dokumentieren zur Nachverfolgung von Kontaktketten
- Einweisung betriebsfremder Personen in die betriebsspezifisch getroffenen Maßnahmen

3. Strategie zum Umgang mit Verdachtsfällen entwickeln

Trotz aller zu treffenden Maßnahmen kann nicht ausgeschlossen werden, dass Infektionen auftreten können. Ein Maßnahmenplan für den Umgang mit Infektionen oder Verdachtsfällen ist daher empfehlenswert.

- Sofern betriebsärztliche Versorgung vorhanden ist, eine einheitliche Notrufnummer definieren und kommunizieren
- Personen mit Verdacht auf Infektion mit MNS/FFP2 ausstatten
- Isolation / Quarantäne der Person(en) in die Wege leiten
- Kontaktpersonen frühestmöglich identifizieren und benachrichtigen
- Bei Bestätigung einer Infektion: Meldung an zuständige Behörde sicherstellen und Person in Quarantäne bzw. Isolation schicken
- Reinigung der Kontaktflächen der betroffenen Person

4. Arbeitsmedizinische Vorsorge und Schutz besonders gefährdeter Personen

Arbeitsmedizinische Vorsorge dient neben der Eindämmung der Infektionsgefahr auch der Abwehr von situationsbedingten Begleiterscheinungen, die aufgrund der pandemischen Ausnahmesituation entstehen können.

- Falls möglich: Beratungsmöglichkeit durch betriebsärztlichen Dienst schaffen (auch anonym)
- Möglichkeit zur Betreuung bei psychosozialen Belastungen durch Veränderung der Arbeitsorganisation und -gestaltung anbieten
- Arbeitsmedizinisches Vorsorgeangebot gewährleisten und ggf. Nachholung ermöglichen
- Anordnung und Durchführung von Quarantänemaßnahmen in Abstimmung mit dem zuständigen Gesundheitsamt und unter Einbezug des betriebsärztlichen Dienstes
- Impfangebot durch betriebsärztlichen Dienst ermöglichen und wenn möglich Impfbereitschaft durch Anreizsetzung erhöhen
- Betriebliche Teststrategie entwickeln
- Ausgabe von Schnell- und/oder Selbsttests (mind. 2 Tests pro Woche)
- Ggf. Testpersonal schulen und mit Schutzkleidung ausstatten

5. Schutzmaßnahmen für Flächen, Bereiche und Einrichtungen, die von Gästen aufgesucht werden

Die folgenden Maßnahmen beziehen sich speziell auf die Bereiche, die von Kunden/Gästen aufgesucht werden und die anders als die meisten Outdoorbereiche mit natürlicher Luftzirkulation einer möglichen Aerosolbelastung unterliegen. In einem Freizeitpark umfasst dies im Detail: Wege vom und zum Parkplatz, Parkein- und Ausgänge, Outdoorbereiche mit hoher Personendichte, Wartebereiche, Attraktionen, Shows, Shops und andere Indoor-Bereiche, Infopoints und -stationen, Gastronomie und Hotellerie, Sanitäranlagen. Allgemein ist der Einsatz von Checklisten für jeden Zuständigkeitsbereich sinnvoll.

5.1 Parkplatz, Parkein- und ausgänge sowie Outdoor-Bereiche mit hoher Personendichte

- Abstandsmarkierungen anbringen (z.B. durch Linien auf dem Boden, Hinweisschilder, Aufkleber)
- Besucherströme lenken durch Einbahnsysteme und Markierungen, ggf. durch mechanische Barrieren
- Kontrollpersonal schulen

5.2 Wartebereiche

- - Abstandsmarkierungen anbringen (z.B. durch Linien auf dem Boden, Hinweisschilder, Aufkleber)
  - dabei auf Nachhaltigkeit und Bestandsfestigkeit achten (Müllreduktion, umweltfreundlicher Farbauftrag, feste Fixierung ohne Rückstände bei Ablösung, Sicherung vor Vandalismus)
  - Wartebereiche falls möglich teilweise oder komplett in den Außenbereich verlegen, andernfalls maximale Durchlüftung ermöglichen und die Aufenthaltsdauer der Gäste durch Besucherlenkung in diesen Bereichen kurzhalten, dabei Gruppenstrukturen beachten
  - Mechanische Barrieren (z.B. Acrylglas) schaffen, falls Abstände nicht eingehalten werden können
  - Maskenpflicht in Wartebereichen einführen und durch Hinweisschilder sowie ggf. den Einsatz von Multimedia darauf verweisen und überwachen
  - Kontrollpersonal schulen und Kontrollintervalle festlegen
  - Reinigungsintervalle von Handläufen, Türdrückern, Drehkreuzen und anderen Kontaktstellen erhöhen und im besten Fall Durchgangssperren durch automatisierte Öffnungssysteme ersetzen
  - Desinfektionsmittelspender leicht zugänglich installieren
  - Sofern möglich: Virtual Queueing oder Zeittickets zur Lenkung der Besucherströme anbieten

5.3 Attraktionen

- Reinigungsplan entwickeln und dokumentieren
- Regelmäßige Reinigung von Kontaktflächen, sofern sie nicht zu Lasten der Kapazität geht. Alternativ Verlängerung der Reinigungsintervalle oder Verzicht, um Wartezeiten zu reduzieren und Auslagerung der Reinigung auf die Nutzer (durch Händedesinfektion vor dem Einstieg etc.)
- Kapazität möglichst maximieren
- Sofern möglich: Gruppenspezifische Besetzung von Attraktionen (auch hier sollte abgewogen werden, inwiefern dadurch die Wartezeit erhöht wird), eventuell durch Vorstrukturierung nach Gruppen in der Warteschlange den Durchlauf optimieren
- Attraktionennutzung nur mit Maske ermöglichen
- Beim Ein- und Ausstieg die Gäste auf Einhaltung der Abstände hinweisen
- Desinfektionsmittelspender am Ein- und Ausgang der Attraktionen installieren
- Attraktionenpersonal (Operators) mit Schutzausrüstung (MNS/FFP2 und ggf. Handschuhen) ausstatten und durch innerbetriebliche Maßnahmen schulen, nach Möglichkeit auf Mehrsprachigkeit des Personals mit direktem Kundenkontakt achten
- Körperkontakte vermeiden

5.4 Shows, Shops und andere Indoor-Bereiche

- - Reinigungsplan entwickeln und dokumentieren
  - Desinfektionsmittelspender an Ein- und Ausgängen installieren
  - Platzierung des Personals organisieren
  - Bestuhlung von Veranstaltungsräumen unter Einhaltung von Mindestabständen festlegen, bzw. freizuhaltende Plätze eindeutig markieren
  - Nutzungsobergrenzen definieren und ggf. durch Anzeigesysteme regulieren
  - Personal (Operators) mit Schutzausrüstung (MNS/FFP2 und ggf. Handschuhen) ausstatten und durch innerbetriebliche Maßnahmen schulen, nach Möglichkeit auf Mehrsprachigkeit des Personals mit direktem Kundenkontakt achten
  - Sofern zutreffend: kontaktloses Bezahlen ermöglichen bzw. Hinweise anbringen, mit denen um die Vermeidung von Barzahlung gebeten wird
  - Kassenbereiche ggf. mit mechanischen Barrieren ausstatten (z.B. Acrylglas)
  - Lüftungsplan umsetzen

5.5 Gastronomie und Hotellerie

- - Reinigungsplan entwickeln und dokumentieren
  - Desinfektionsmittelspender installieren
  - In Wartebereichen Markierungen und Hinweisschilder anbringen
  - In Restaurants Platzierung durch Personal organisieren
  - Bestuhlung unter Einhaltung von Mindestabständen festlegen
  - Sofern Selbstbedienung: Personenströme regulieren (z.B. durch Wegerichtungen / Einbahnstraßen) oder vorab festgelegte Speisezeiten
  - Einweg-Handschuhe bei Selbstbedienung ausgeben
  - Kapazität maximieren
  - Servicepersonal mit Schutzausrüstung (MNS/FFP2 und ggf. Handschuhen) ausstatten und durch innerbetriebliche Maßnahmen schulen, nach Möglichkeit auf Mehrsprachigkeit des Personals mit direktem Kundenkontakt achten
  - Kontaktloses Bezahlen ermöglichen bzw. Hinweise anbringen, mit denen um die Vermeidung von Barzahlung gebeten wird
  - Kassenbereiche ggf. mit mechanischen Barrieren ausstatten (z.B. Acrylglas)
  - Hotelzimmer: Reinigungsplan erweitern (z.B. angepasste Reinigungsmittel nutzen, Flächendesinfektion, Hygienesiegel)
  - Lüftungsplan umsetzen

5.6 Sanitäranlagen

- Reinigungsplan entwickeln und dokumentieren
- Desinfektionsmittelspender installieren
- Ggf. zusätzliche Möglichkeiten zur Handreinigung anbieten
- Ggf. Sensorsteuerung von Seifenspendern, Wasserhähnen, Trocknungsanlagen sowie Toilettenspülungen ermöglichen
- Nutzungsobergrenzen definieren und ggf. durch automatisierte Anzeigesysteme regulieren
- Ggf. Wegerichtungen definieren bzw. Einbahnstraßen einrichten
- Lüftungsplan umsetzen

6. Abstimmung mit dem örtlichen Gesundheitsamt

- - Hygienepläne und Maßnahmenkataloge sollten in Abstimmung mit dem zuständigen Gesundheitsamt entwickelt und koordiniert werden
  - Aktualisierung und Anpassung der Maßnahmen an die jeweils gültige Corona-Verordnung von Land und Bund

7. Evaluation der getroffenen Maßnahmen

- - Kontinuierlicher Einbezug der Perspektive der Gäste zur Evaluation der Maßnahmen und Überprüfung ihrer Akzeptanz
  - Hohe Reaktionsbereitschaft und agiles Management zur flexiblen Anpassung an kritische Rückmeldungen der Befragten etablieren

Recommended action plan for the safe opening and operation of amusement parks under pandemic conditions

1. Risk assessment of the site

In the context of the pandemic situation, a systematic hazard assessment should be conducted of all relevant areas with respect to risk of infection.

Relevant Hazard Areas:

- Indoor areas:

- Waiting and exit areas

- Catering / restaurants

- Sanitary facilities

- Stores

- Indoor attractions and shows

- Outdoor areas:

- Routes with a high volume of people, e.g. central plazas, intersections, entrances and exits

- Internal areas:

- Stores

- Offices

- Service areas

- Canteens and break rooms

2. Development of a company pandemic plan for occupational health and safety

The additional infection control measures required at the workplace should be recorded centrally in a company hygiene concept and implemented systematically using a task force. In general, all measures must be communicated comprehensively and the corresponding instructions must be standardized. In the case of international personnel, care must be taken to ensure that the information material is multilingual in order to guarantee accessibility.

Possible in-house measures:

- Ensure that the safety distance among employees is maintained.
- Avoid touching (through instructions for action)
- Establish coughing, sneezing and hygiene etiquette and communicate accordingly
- Create sanitary infrastructure (water, soap, sanitizing facilities, disposable towels)
- Make mouth-nose protection (MNP) and FFP2 masks available to employees
- Formulate instructions for correct wearing of MNP/FFP2 (in words and pictures)
- Establish ventilation concept and, if necessary, set up room air cleaners.
- Ensure maintenance cycles for ventilation systems
- Avoid operation of ventilation systems without filtration
- Organize multiple occupancy of rooms and define room occupancy if necessary
- Divide staff into defined work groups to reduce internal contacts and to keep the number of people affected by quarantine low in the event of infection within the group.
- If feasible: allow home office
- Reduce company-related contacts in rooms with a high density of people (canteen, elevators, corridors).
- If contact with people cannot be avoided despite operational measures, install signs and markings
- Reduce the number of people per room (upper limit 10 square meters per person).
- In areas where the minimum distance of 1.5 m cannot be maintained, mechanical barriers (e.g., acrylic glass) should be created (e.g., at issues, cash registers, help desks)
- Install distance markings in areas where queuing can occur (e.g. canteen)
- Establish action plan for dealing with customers and in the field
- Reduce business travel and use online and teleconferencing tools where possible
- Use work equipment and tools in a person-related manner and increase cleaning intervals
- Work time management (if necessary, introduce shift work to reduce internal personal contacts)
- Document contact data and contact time of external persons to track contact chains
- Instruction of external persons in the measures taken for specific companies

3. Development of a strategy for dealing with suspicious cases

Despite all the measures to be taken, it cannot be ruled out that infections may occur. A plan of action for dealing with infections or suspected cases is therefore recommended.

- If company medical care is available, define and communicate a uniform emergency telephone number
- Provide persons with suspected infection with MNP/FFP2
- Initiate isolation / quarantine of the person(s)
- Identify and notify contacts as soon as possible
- If infection is confirmed: ensure notification to competent authority and send person to quarantine
- Clean the contact surfaces of the affected person

4. Occupational health care and protection of persons at particular risk

In addition to containing the risk of infection, preventive occupational health care also serves to ward off situational side effects that may arise as a result of the exceptional pandemic situation.

- If possible, provide counseling by the company medical service (also anonymous)
- Offer the possibility of support in the event of psychosocial stress due to changes in work organization and design
- Ensure preventive occupational health care and, if necessary, make it possible to catch up on it
- Arrangement and implementation of quarantine measures in coordination with the responsible health office and with the involvement of the company medical service
- Enable the company medical service to offer vaccinations and, if possible, increase the willingness to vaccinate by setting incentives
- Develop company testing strategy
- Issue rapid and/or self-tests (at least 2 tests per week)
- If necessary, train test personnel and equip them with protective clothing

5. Protective measures for areas, areas and facilities frequented by guests

The following measures relate specifically to areas frequented by customers/guests and which, unlike most outdoor areas with natural air circulation, are subject to possible aerosol exposure. In an amusement park, this includes in detail: routes to and from the parking lot, park entrances and exits, outdoor areas with a high density of people, waiting areas, attractions, shows, stores and other indoor areas, info points and stations, food and beverage outlets and hotels, sanitary facilities. In general, the use of checklists for each area of responsibility is useful.

5.1 Parking lot, parking entrances and exits, and outdoor areas with a high density of people

- Install distance markings (e.g., by lines on the ground, signs, stickers, etc.)
- Direct visitor flows through one-way systems and markings, if necessary through mechanical barriers
- Train control personnel

5.2 Waiting areas

- Install distance markings (e.g., lines on the floor, information signs, stickers, etc.)
- pay attention to sustainability and durability (waste reduction, environmentally friendly paint application, firm fixation without residues when removed, protection against vandalism)
- If possible, relocate waiting areas partially or completely outdoors, otherwise allow for maximum ventilation and keep the length of stay of guests short by directing visitors to these areas, paying attention to group structures
- Create mechanical barriers (e.g. acrylic glass) if distances cannot be maintained
- Introduce mandatory masks in waiting areas and use signs and, if necessary, multimedia to point this out and monitor the situation
- Train control personnel and define control intervals
- Increase cleaning intervals for handrails, door handles, turnstiles and other contact points and, in the best case, replace passage barriers with automated opening systems
- Install disinfectant dispensers that are easily accessible
- If possible, offer virtual queueing or timed tickets to direct visitor flows

5.3 Attractions

- Develop and document cleaning schedule
- Regular cleaning of contact surfaces, provided it is not detrimental to capacity. Alternatively, extend cleaning intervals or waive them to reduce wait times and outsource cleaning to users (by sanitizing hands before entry, etc.)
- Maximize capacity as much as possible
- If possible: group-specific staffing of attractions (here, too, the extent to which this increases waiting times should be weighed up), possibly optimize throughput by pre-structuring according to groups in the queue
- Allow use of attractions only with mask
- When entering and exiting the attraction, point out to guests that they must keep their distance from the attraction
- Install disinfectant dispensers at the entrance and exit of attractions
- Equip attraction personnel (operators) with protective equipment (MNS/FFP2 and, if necessary, gloves) and train them through in-house measures; if possible, ensure that personnel with direct customer contact are multilingual
- Avoid physical contact

5.4 Shows, stores and other indoor areas

- Develop and document cleaning schedule
- Install disinfectant dispensers at entrances and exits
- Organize placement of personnel
- Determine seating in event rooms in compliance with minimum distances, or clearly mark seats to be kept free
- Define upper usage limits and, if necessary, regulate them by means of display systems
- Equip personnel (operators) with protective equipment (MNP/FFP2 and gloves, if necessary) and train them through in-house measures; if possible, ensure that personnel with direct customer contact are multilingual
- If applicable: enable contactless payment or post notices asking customers to avoid paying in cash
- If necessary, equip checkout areas with mechanical barriers (e.g., acrylic glass)
- Implement ventilation plan

5.5 Catering and hotel business

- Develop and document cleaning plan
- Install sanitizer dispensers
- Install markings and signs in waiting areas
- In restaurants, organize placement by staff
- Determine seating in compliance with minimum distances
- If self-service: regulate flow of people (e.g., through directions / one-way streets) or pre-determined dining times
- Issue disposable gloves for self-service
- Maximize capacity
- Equip service personnel with protective equipment (MNP/FFP2 and gloves if necessary) and train them through in-house measures; if possible, ensure that personnel with direct customer contact are multilingual
- Enable contactless payment or post notices asking customers to avoid paying in cash
- If necessary, equip checkout areas with mechanical barriers (e.g., acrylic glass)
- Hotel rooms: extend cleaning plan (e.g. use adapted cleaning agents, surface disinfection, hygiene seal)
- Implement ventilation plan

5.6 Sanitary facilities

- Develop and document cleaning plan
- Install disinfectant dispensers
- If necessary, offer additional options for hand cleaning
- If necessary, enable sensor control of soap dispensers, faucets, drying systems and toilet flushes
- Define upper usage limits and, if necessary, regulate them by means of automated display systems
- If necessary, define directions of travel or set up one-way streets
- Implement ventilation plan

6. Coordination with the local health office

- Hygiene plans and catalogues of measures should be developed and coordinated in consultation with the responsible health office
- Updating and adaptation of the measures to the respectively valid Corona ordinance of the state and federal government

7. Evaluation of the measures taken

- Continuous inclusion of the guests' perspective to evaluate the measures and check their acceptance by guest monitoring
- Establish high responsiveness and agile management to flexibly adapt to critical feedback from respondents
